# Accuracy Analysis of Holes Drilled in Ductile Cast Iron with an HSS Helical Drill Bit

**DOI:** 10.3390/ma19122606

**Published:** 2026-06-17

**Authors:** Radosław Sójka, Piotr Ziarkowski, Kamil Klamczyński, Natalia Kowalska, Slawomir Blasiak, Lukasz Nowakowski, Michal Skrzyniarz

**Affiliations:** Department of Manufacturing Engineering and Metrology, Kielce University of Technology, al. Tysiąclecia Państwa Polskiego 7, 25-314 Kielce, Poland; pziarkowski@tu.kielce.pl (P.Z.); kklamczynski@tu.kielce.pl (K.K.); nkowalska@tu.kielce.pl (N.K.); sblasiak@tu.kielce.pl (S.B.); lukasn@tu.kielce.pl (L.N.); mskrzyniarz@tu.kielce.pl (M.S.)

**Keywords:** ductile cast iron, HSS twist drill, cylindricity error, roundness deviation, dry machining, process dynamics

## Abstract

Controlling macro-geometrical errors in the dry drilling of ductile cast iron remains a critical challenge for sustainable and cost-efficient automotive component manufacturing. This paper investigates the influence of cutting speed (v_c_) and feed per revolution (f_n_) on the dimensional and shape accuracy of holes drilled in EN-GJS-500-7 ductile cast iron using an HSS DIN 338 helical drill (Ø 11.8 mm, Ceratizit) on an AVIA VMC800 CNC milling centre. A one-factor-at-a-time (OFAT) experimental design was applied: the feed effect was evaluated at v_c_ = 10 m/min with f_n_ ∈ {0.10, 0.15, 0.20} mm/rev, while the speed effect was evaluated at f_n_ = 0.20 mm/rev with v_c_ ∈ {10, 25, 30} m/min. Cutting forces, torques, and vibration accelerations were recorded using an HBM MSC 10 transducer and a PCB 356A01 tri-axial accelerometer. Hole geometry was assessed on a Zeiss Contura G2 coordinate-measuring machine (CMM), and surface texture was evaluated with a TOPO 01P contact profilometer. The expanded measurement uncertainty (k = 2) was estimated based on duplicate test specimens. All drilled holes fell within the IT12 dimensional tolerance (PN-EN 22768-1:1999 grade c), with diameter oversizes ranging from +0.26 mm to +0.46 mm relative to the nominal bore. Cutting speed was identified as the dominant factor affecting both diameter oversize and cylindricity, which increased by 60% (from 0.10 to 0.16 mm) as v_c_ rose from 10 to 30 m/min. Vibration accelerations increased nonlinearly between v_c_ = 25 and 30 m/min (by a factor of 2.5×), indicating an approach to a structural resonance condition. The lowest surface roughness (R_a_ = 6.6 µm) was obtained at v_c_ = 25 m/min. These findings establish clear physical baselines for tool deflection limits, demonstrating that managing dynamic process stability is vital for optimising macro-geometrical accuracy in the dry machining of cast iron alloys.

## 1. Introduction

Drilling is among the most widely used metal-cutting operations, accounting for approximately 33% of all machining processes in manufacturing [1,2,3,4]. Despite the proliferation of advanced solid-carbide and coated drill designs, high-speed steel (HSS) tools remain in routine industrial use due to their lower cost, sufficient toughness for interrupted cuts, and ease of re-sharpening [5]. The drilling process is characterised by confined chip evacuation, limited tool visibility during cutting, and a non-uniform chip-load distribution along the cutting edge—factors that collectively make the dimensional and shape control of the produced hole more challenging than in turning or milling [6,7,8,9].

Key process parameters affecting hole quality include cutting speed (v_c_), feed rate (f_n_), tool geometry (point angle, helix angle, and web thickness), workpiece material properties, and the cooling/lubrication strategy [1,2,10,11,12]. For any given tool–material combination, the diameter oversize, circularity deviation, cylindricity error, and surface roughness of the hole are sensitive functions of the selected cutting conditions [5,8,9,13,14].

Ductile cast iron (DCI), standardised under the EN 1563 family, is increasingly specified in the automotive and aerospace sectors as a substitute for wrought steel in structural components such as crankshafts, steering knuckles, and wheel hubs, owing to its combination of high tensile strength (≥500 MPa for grade EN-GJS-500-7), good impact resistance, excellent castability, and approximately 10% lower density than steel [15,16]. The ferritic-pearlitic matrix with spheroidal graphite nodules gives EN-GJS-500-7 a machinability rating broadly comparable to low-alloy steel [17].

However, the periodic transition of the cutting edge between the hard matrix and the soft graphite nodules creates specific mechanical anisotropy [18] and micro-fluctuations in cutting forces [19]. These structural variations produce unique abrasive wear and thermal effects distinct from those observed in grey iron or carbon steel [15,16,17,20].

A survey of the recent literature reveals that drilling in cast iron alloys has remained an active area of research, though the focus is shifting towards advanced tool designs and monitoring. For instance, Zhang et al. [19,21] investigated the wear mechanisms of micro-textured drill tools during the single-factor drilling of compacted graphite iron (CGI). Regarding process conditions, Zhu et al. [22] analysed the spatial temperature distribution along the drill edge, showing a severe heat concentration near the tool centre during the high-throughput dry drilling of cast iron. Furthermore, Kakino [23] emphasised the importance of process stability and torque monitoring to prevent tool failure in cast iron machining.

Tusset et al. [20] investigated carbide twist drills in the dry drilling of grey cast iron and reported that the microgeometry of the cutting edge strongly influenced both the surface roughness and dimensional accuracy. Klocke et al. [15] and Meena and El Mansori [16] focused on ADI with carbide tools, reporting that the high silicon and retained austenite content substantially accelerated flank wear.

However, while these modern studies [15,17,19,20,22] predominantly focus on advanced tool modifications, solid carbide tooling, or alternative cast iron grades (such as CGI or ADI), systematic data regarding the direct impact of cutting parameters on macro-geometrical errors for conventional HSS tools remain sparse. No equivalent study for the HSS drilling of EN-GJS-500-7 under varied cutting-speed and feed conditions has been identified in the open literature, establishing the motivation for the present work.

The aim of this study is to quantify the influence of the cutting speed and feed per revolution on the dimensional accuracy (diameter oversize, circularity, cylindricity, and straightness) and surface roughness of holes drilled in EN-GJS-500-7 ductile cast iron with a standard HSS DIN 338 helical drill, and to provide an estimate of the associated measurement uncertainty.

## 2. Materials and Methods

The experimental programme was designed to isolate and quantify the individual effects of two key process parameters—cutting speed (v_c_) and feed per revolution (f_n_)—on the quality of holes drilled in EN-GJS-500-7 ductile cast iron. Both parameters were varied within ranges representative of industrial HSS drilling practice for cast iron, based on the tool manufacturer’s recommendations and data reported in the literature [1,5,20]. The tests were conducted under dry cutting conditions, without the application of cutting fluid or minimum quantity lubrication, in order to replicate conditions frequently encountered in foundry and automotive component machining where coolant supply to deep holes is impractical. All specimens were prepared from the same certified EN-GJS-500-7 bar stock to minimise inter-specimen variability in workpiece properties. A single drill of the same catalogue specification was used throughout the entire test series to eliminate tool-to-tool geometric variation as a source of scatter.

### 2.1. Experimental Design

A one-factor-at-a-time (OFAT) experimental design was adopted with two independent series. The feed series (v_c_ = 10 m/min, constant) evaluated f_n_ = 0.10 mm/rev (specimen 3), 0.15 mm/rev (specimen 2), and 0.20 mm/rev (specimen 1), isolating the effect of feed rate at the lowest tested cutting speed. The speed series (f_n_ = 0.20 mm/rev, constant) covered v_c_ = 10 m/min (specimen 1), 25 m/min (specimen 5), and 30 m/min (specimen 6), isolating the effect of cutting speed at the highest tested feed rate. Specimen 4 was drilled under identical conditions to specimen 1 (v_c_ = 10 m/min, f_n_ = 0.20 mm/rev) to provide an estimate of process and measurement repeatability, which forms the basis of the uncertainty analysis in Section 2.6. The full parameter matrix is given in Table 1.

While statistical DoE methods are often used for optimisation, they can obscure the direct physical relationships because of data averaging. In this study, the OFAT approach was used to isolate how individual cutting parameters affect roundness and cylindricity errors. This approach helps to identify clear thresholds for chip crowding and tool deflection, and it is widely used in recent studies on drilling cast iron alloys [19].

### 2.2. Tool Characteristics

**Figure 1:**
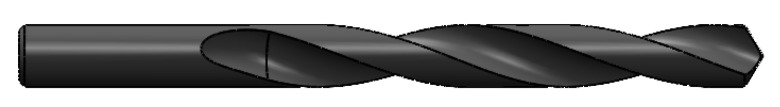
HSS helical drill Ø11.8 mm manufactured by Ceratizit.

A helical drill bit with diameter Ø 11.8 mm, manufactured from HSS DIN 338 steel by Ceratizit (Mamer, Luxembourg; catalogue designation N.11.80.R.5D.DIN338 VAP) (Figure 1), was used throughout the trials. The drill has a standard 118° point angle and two straight flutes. Full tool specifications are listed in Table 2.

### 2.3. Workpiece Material

Cylindrical specimens (Ø 39 mm × 30 mm) were machined from continuously cast EN-GJS-500-7 ductile cast iron bar stock obtained from a certified commercial supplier in the raw, un-heat-treated as-cast condition, establishing a standard hole aspect ratio of L/D ≈ 2.54. The material microstructure consists of approximately 50% pearlite, 40% ferrite, and 10% spheroidal graphite. Chemical composition is given in Table 3. Key properties relevant to machinability include elastic modulus ≈ 170 GPa; tensile strength ≥ 500 MPa; Brinell hardness 170–230 HBW; low thermal conductivity (≈33 W/m·K); and relatively poor vibration damping capacity compared with grey iron, so that machining-induced dynamic excitation is not rapidly attenuated through the workpiece [15].

Specimens were clamped in an Axon K72-125 four-jaw chuck with hard jaws (Kunshan Omatei Mechanical and Electrical Equipment Co., LTD, Kunshan, China). A torque wrench was used to apply a consistent tightening torque of 10 Nm to all specimens, ensuring repeatable clamping force.

### 2.4. Test Rig and Instrumentation

Cutting forces (*Fx*, *Fy*, and *Fz*) and torques (*Mx*, *My*, and *Mz*) were recorded continuously during each drilling cycle using an HBM MSC 10 multi-axis force and torque transducer, connected to an MX840B signal conditioning amplifier operated via Catman Easy acquisition software (version 3.4, Hottinger Brüel & Kjær, Darmstadt, Germany). Vibration accelerations (ax, ay, and az) were acquired with a PCB 356A01 tri-axial accelerometer (PCB Piezotronics, Depew, NY, USA) mounted on the machine spindle housing. Each recorded signal was averaged over 1000 consecutive samples to reduce high-frequency noise while preserving the physically meaningful force envelope.

### 2.5. Machine Tool

**Figure 2:**
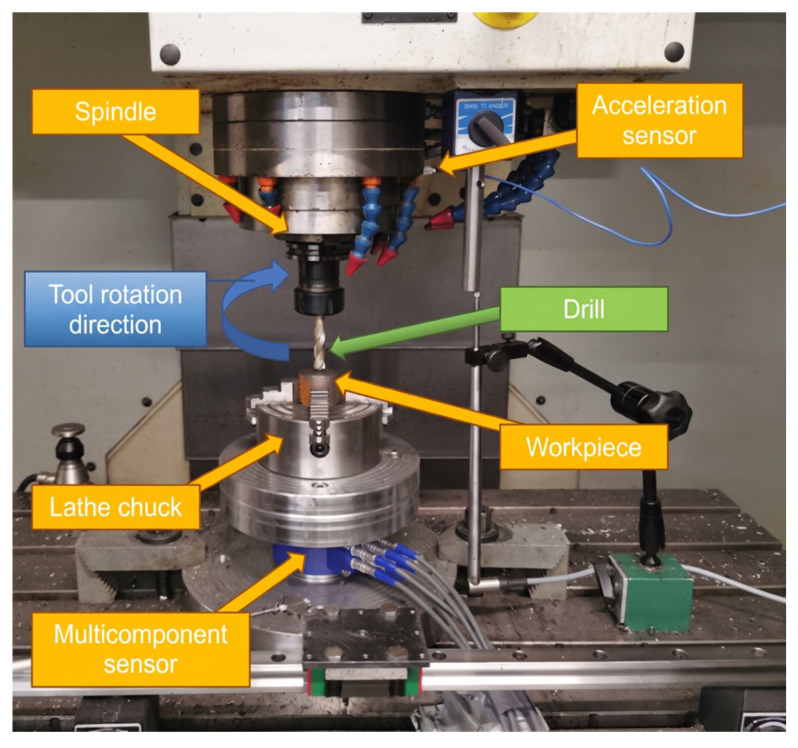
AVIA VMC800 machining centre with attached test bench. Components labelled: spindle, acceleration sensor, drill, workpiece, lathe chuck, multicomponent sensor, and tool rotation direction.

The cutting trials were performed on an FOP AVIA VMC800 vertical CNC machining centre (FOP AVIA S.A., Warsaw, Poland) (Figure 2). The machine features a cross table moving in the X–Y plane and a vertical quill on a fixed column (Z axis), with an optional fourth CNC rotary A-axis and a 24-position drum tool magazine.

### 2.6. Measurement Equipment and Measurement Uncertainty

Hole geometry was measured on a Zeiss Contura G2 coordinate-measuring machine (CMM; Carl Zeiss AG, Oberkochen, Germany) using a ruby-ball stylus. Five circular cross-sections were evaluated at depths of 5, 10, 15, 20, and 25 mm from the specimen face. Circularity was assessed at each cross-section; cylindricity was computed over the entire measured length (5–25 mm); and straightness was evaluated on four axial generators spaced 90° apart.

Surface texture was assessed with a TOPO 01P contact profilometer using a 2 µm radius tip, a 0.8 mm Gaussian filter cut-off, and a 5 mm evaluation length, in accordance with ISO 4288:1996 [24].

Measurement uncertainty was estimated following the GUM framework (ISO/IEC Guide 98-3). Two dominant uncertainty sources were considered: (i) instrument repeatability and (ii) process repeatability (hole-to-hole variation under constant conditions), derived from the duplicate specimen pair (specimens 1 and 4). The expanded uncertainty UR = k·uc was computed with k = 2 (approximately 95% confidence). Results are summarised in Table 4.

## 3. Results

### 3.1. Cutting Forces, Torques, and Accelerations

**Figure 3:**
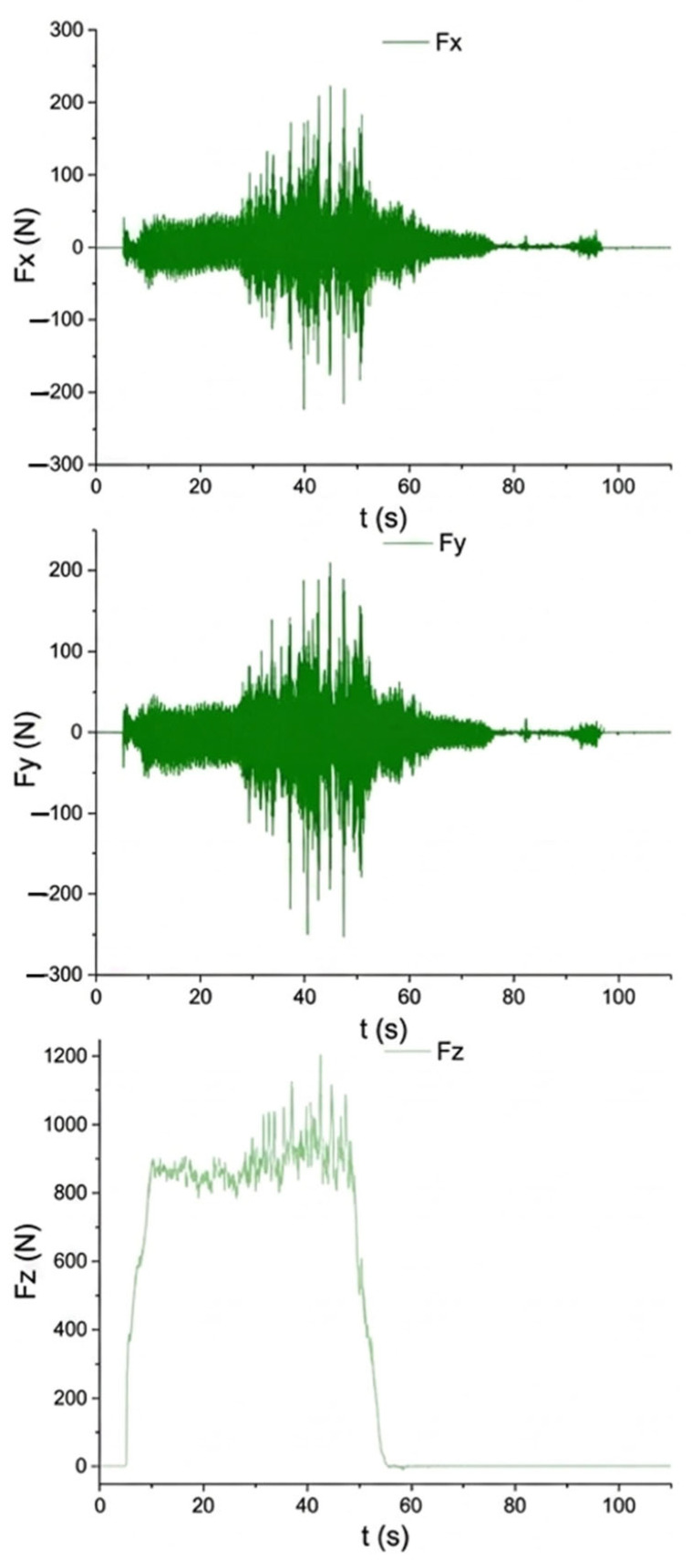
Time history of force components (*Fx*, *Fy*, and *Fz*) obtained during the drilling process for specimen 1.

**Figure 4:**
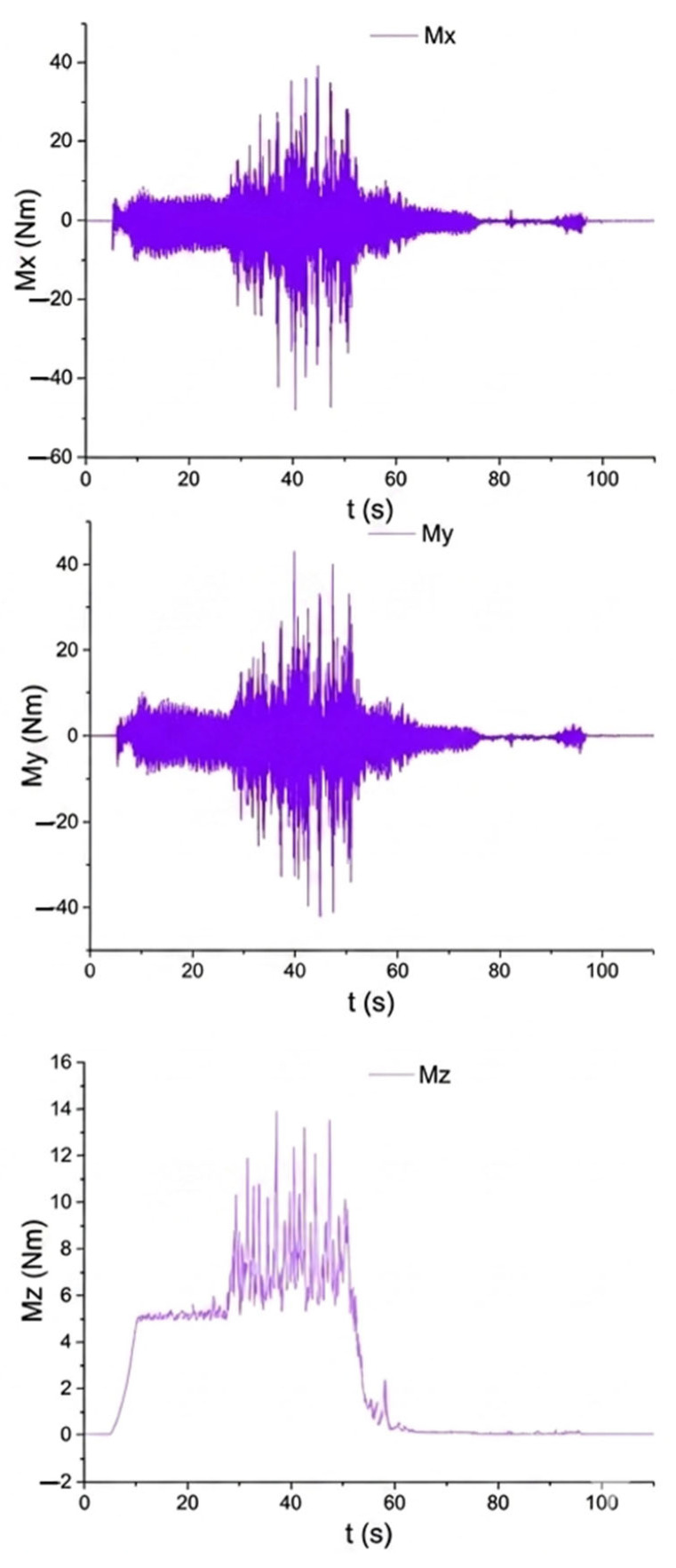
Time history of bending moments (*Mx* and *My*) and torque (*Mz*) obtained during the drilling process for specimen 1.

**Figure 5:**
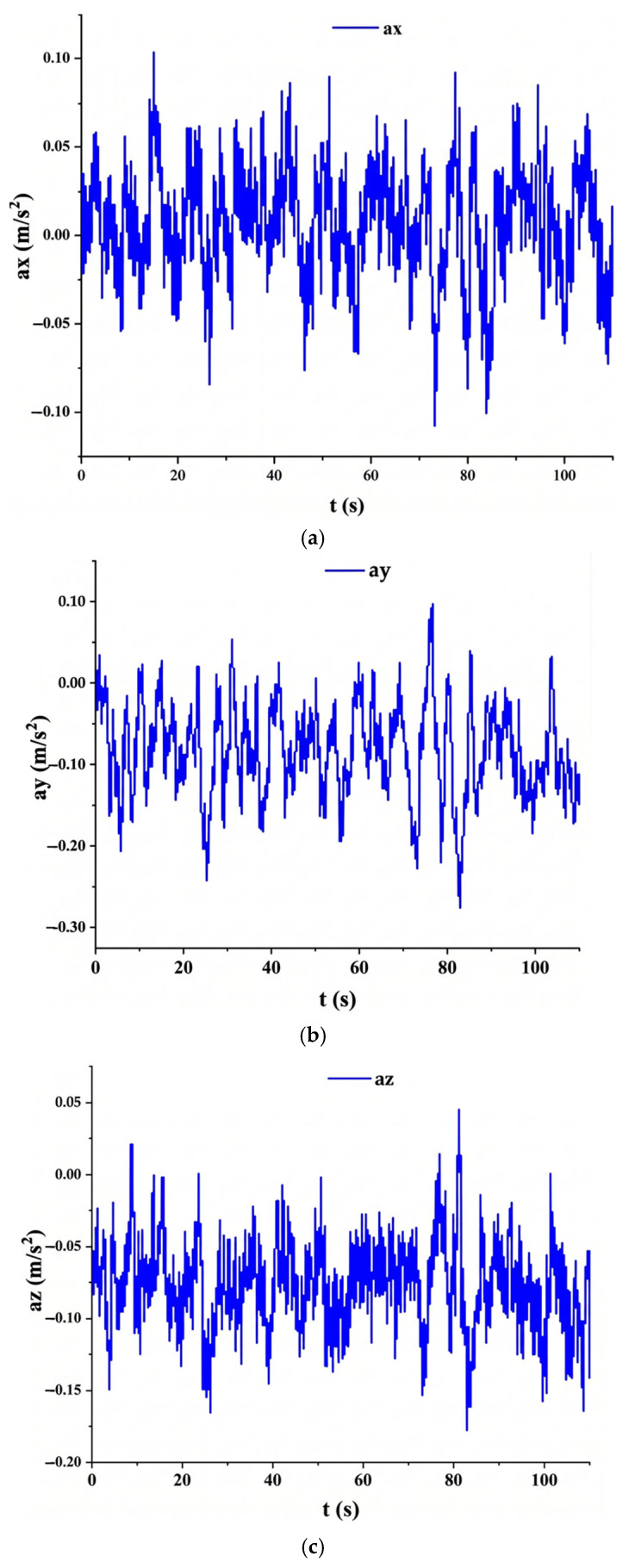
Vibration acceleration diagrams (ax, ay, and az) obtained during drilling for specimen 1: (**a**) acceleration plot ax (X-axis); (**b**) acceleration plot ay (Y-axis); and (**c**) acceleration plot az (Z-axis).

Representative time-history plots of forces (Figure 3), torques (Figure 4), and accelerations (Figure 5) are shown for specimen 1 (v_c_ = 10 m/min, f_n_ = 0.20 mm/rev). The axial force *Fz* exhibits the characteristic transient-and-plateau profile: it rises from zero during chisel-edge engagement, reaches a steady-state plateau of approximately 800–900 N, and peaks at ~1200 N during the final breakthrough phase (~t = 40–55 s). Vibration accelerations at this condition are very low (peak |a| < 0.10 m/s^2^), confirming quasi-static drilling dynamics at v_c_ = 10 m/min.

Figure 3 Force diagrams (*Fx*, *Fy*, and *Fz*) and Figure 4 Torque diagrams (*Mx, My,* and *Mz*) obtained during the drilling process for specimen 1 (v_c_ = 10 m/min, f_n_ = 0.20 mm/rev).

The maximum and minimum values of all force, torque, and acceleration components for all six specimens are compiled in Table 5 and visualised in Figure 6, Figure 7, Figure 8, Figure 9, Figure 10, Figure 11, Figure 12, Figure 13 and Figure 14.

The axial force *Fz* increases strongly with the cutting speed, 1213 N (v_c_ = 10 m/min), 1650 N (v_c_ = 25 m/min), and 1652 N (v_c_ = 30 m/min)—an increase of approximately 36% from v_c_ = 10 to v_c_ = 25 m/min, with no further increase at v_c_ = 30 m/min. The drilling torque *Mz* increases by 82% from v_c_ = 10 (14.6 Nm) to v_c_ = 25 m/min (26.5 Nm), and then remains virtually unchanged at v_c_ = 30 m/min (26.1 Nm), indicating a transition to a thermally softened cutting regime.

The most significant finding concerns vibration accelerations. At v_c_ = 10 m/min, all amplitudes are below 27 m/s^2^. At v_c_ = 25 m/min, they rise to 54/36/42 m/s^2^ in X/Y/Z. At v_c_ = 30 m/min, they jump to 131/95/101 m/s^2^—an increase of 2.4–2.6× relative to v_c_ = 25 m/min and 5–6× relative to v_c_ = 10 m/min. This sharp escalation suggests that the drill–spindle system approaches a structural resonance condition, and directly explains the disproportionate degradation in hole cylindricity at v_c_ = 30 m/min.

The duplicate specimens 1 and 4 (identical parameters) exhibit markedly different force values (*Fz*_*max*: 1213 vs. 1567 N; and *Fx*_*max*: 211 vs. 324 N—differences of +29% and +54%). This genuine process variability reinforces the need for repeated measurements in future experimental designs.

Furthermore, the intense thermo-mechanical loading inherent to dry drilling operations significantly impacts the subsurface integrity of the hole wall. Within the severely plastically deformed and thermally affected zone directly adjacent to the cutting area, the combination of high localised cutting temperatures and severe shear strains induces microstructural distortion and localised work hardening. This mechanism is accompanied by minor plastic deformation and the directional elongation of the spheroidal graphite nodules along the cutting path, particularly under the high-throughput conditions of v_c_ = 30 m/min where the thermal softening of the matrix accelerates subsurface displacement.

### 3.2. Surface Roughness

**Figure 15:**
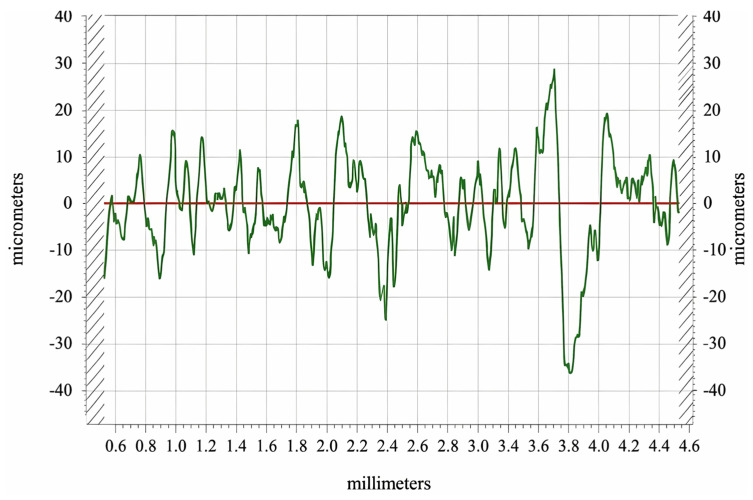
Roughness profile in specimen hole 1. The green line represents the measured roughness profile, and the red line indicates the mean line.

**Figure 16:**
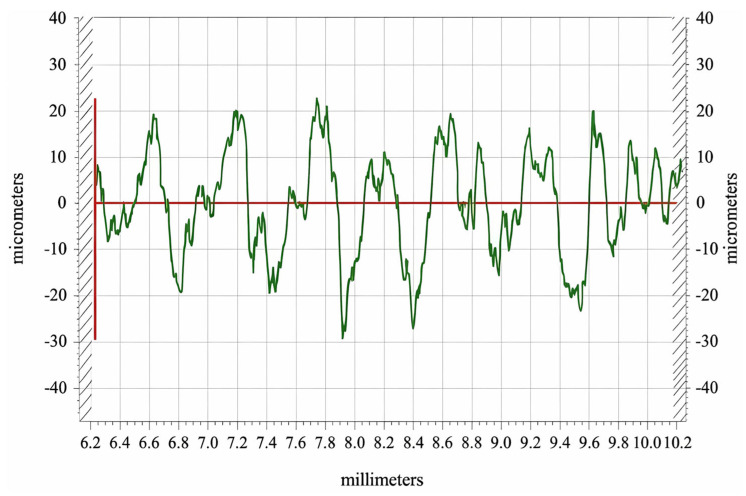
Roughness profile in specimen hole 2. The green line represents the measured roughness profile, and the red line indicates the mean line.

**Figure 17:**
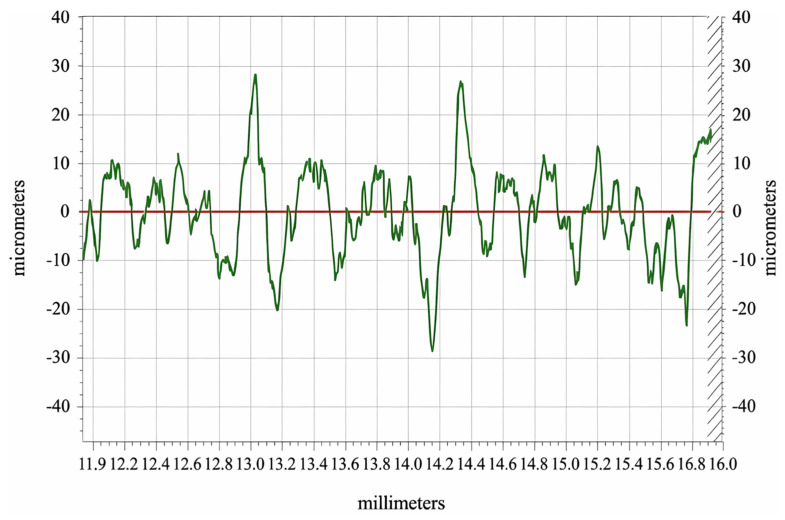
Roughness profile in specimen hole 3. The green line represents the measured roughness profile, and the red line indicates the mean line.

**Figure 18:**
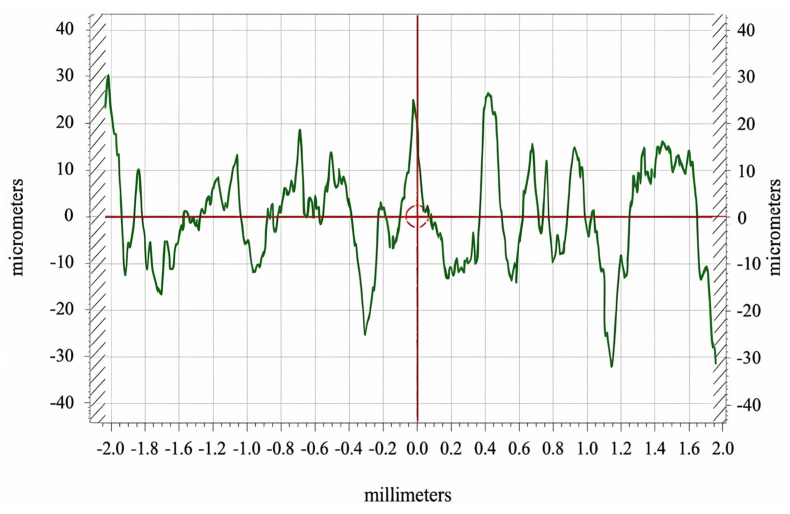
Roughness profile in specimen hole 4. The green line represents the measured roughness profile, and the red line indicates the mean line.

**Figure 19:**
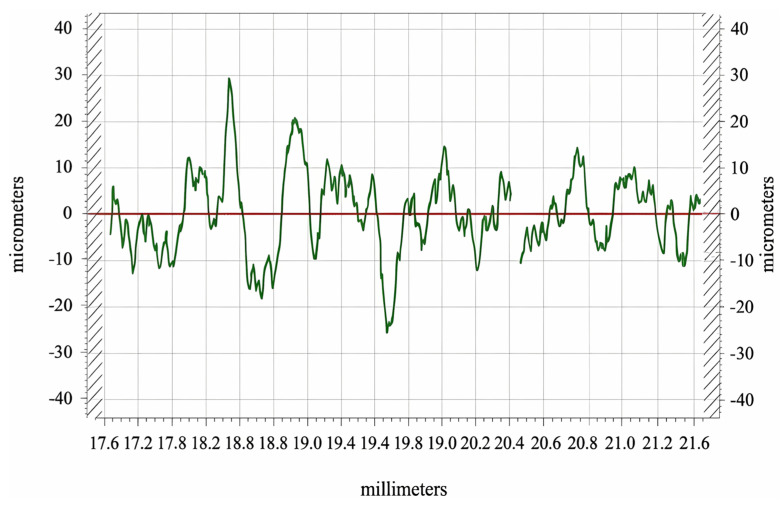
Roughness profile in specimen hole 5. The green line represents the measured roughness profile, and the red line indicates the mean line.

**Figure 20:**
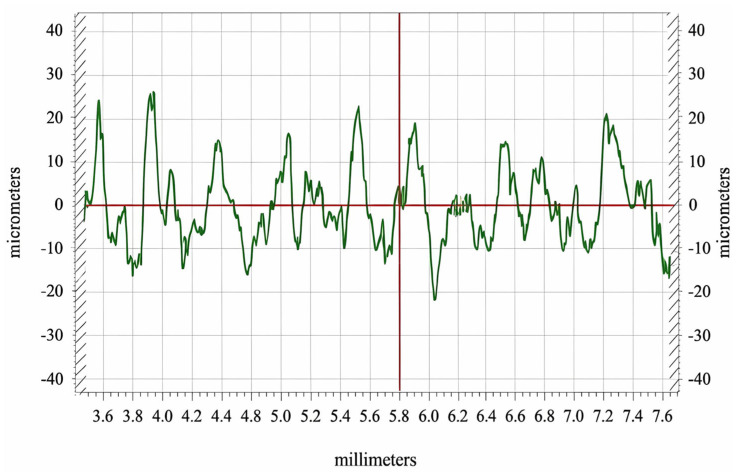
Roughness profile in specimen hole 6. The green line represents the measured roughness profile, and the red line indicates the mean line.

Roughness profiles measured on the bore surface of each specimen (Figure 15, Figure 16, Figure 17, Figure 18, Figure 19 and Figure 20) were evaluated for seven ISO 4287 parameters. The results are summarised in Table 6.

The effect of the feed rate (v_c_ = 10 m/min constant): The R_a_ values are 7.2, 9.1, and 7.5 µm for f_n_ = 0.10, 0.15, and 0.20 mm/rev, respectively. The non-monotonic relationship (specimen 2 with the highest R_a_) is within the measurement uncertainty of ±0.92 µm. R_sk_ approaches zero for all specimens in the feed series (0.02–0.70), indicating approximately symmetrical height distributions typical of freshly drilled surfaces.

The effect of the cutting speed (f_n_ = 0.20 mm/rev constant): The Ra values are 7.5, 6.6, and 7.2 µm for v_c_ = 10, 25, and 30 m/min. The minimum R_a_ at v_c_ = 25 m/min (−12% vs. v_c_ = 10 m/min) is consistent with the improved chip formation regularity at intermediate speeds [15,17]. The slight increase at v_c_ = 30 m/min may reflect thermal effects or chatter onset.

All specimens exhibited R_a_ in the range of 6.6–9.1 µm, corresponding to the surface roughness class N9 (ISO 1302) [25], typical of the as-drilled condition without reaming.

### 3.3. Dimensional Accuracy—Hole Diameter

Hole diameters were measured at five axial positions and a best-fit cylinder was computed over the full measurement range. The results are given in Table 7.

All holes exhibit a positive diameter oversize ranging from +0.21 mm (specimen 1, depth 25 mm) to +0.57 mm (specimen 6, depth 10 mm). Specimen 6 (v_c_ = 30 m/min) exceeds tolerance grade c at the depths of 5 and 10 mm, placing those positions in grade v (very coarse, ±1.0 mm). All remaining measurements fall within grade c.

Cutting speed has a clear monotonic positive effect on cylinder oversize: +0.26 mm at 10 m/min, +0.37 mm at 25 m/min, and +0.46 mm at 30 m/min—a 77% increase exceeding the measurement uncertainty (UR = ±0.07 mm). A consistent diameter taper is observed in all specimens: the diameter is largest at entry (5 mm depth) and decreases progressively to the smallest value at a 25 mm depth, typical of the transient drilling phase.

### 3.4. Shape Accuracy—Circularity, Cylindricity, and Straightness

Circularity deviations (Table 8) are uniformly small across all specimens (0.01–0.05 mm) and show no clear dependence on either the cutting speed or feed within the tested range, indicating that radial form errors in individual cross-sections are well-controlled regardless of cutting conditions.

Cylindricity (Table 8) shows a clear positive correlation with cutting speed: 0.10 mm (v_c_ = 10 m/min), 0.12 mm (v_c_ = 25 m/min), and 0.16 mm (v_c_ = 30 m/min)—a 60% increase. This speed-dependent trend is statistically meaningful relative to UR = ±0.021 mm. Within the feed series, the cylindricity ranges from 0.07 to 0.11 mm with no consistent trend, falling within the uncertainty band.

The mean straightness (Table 9) deviation increases with cutting speed (0.045 mm at 10 m/min, 0.055 mm at 25 m/min, and 0.060 mm at 30 m/min) but varies non-monotonically with the feed, suggesting localised deflection events not systematically controlled by either parameter in the range tested.

## 4. Discussion

The present results are broadly consistent with the limited existing data on the drilling of cast iron with HSS tools. Tusset et al. [20] reported diameter oversizes of 0.10–0.35 mm when drilling grey cast iron with carbide twist drills at comparable feed rates, noting a positive correlation between the cutting speed and bore oversize—a trend confirmed in the present study for DCI. The larger oversizes observed here (up to +0.57 mm) likely reflect the higher elastic modulus and reduced damping of EN-GJS-500-7 compared with grey iron, which may amplify drill deflection and dynamic runout.

The finding that cutting speed exerts a stronger effect on cylindricity than feed rate has practical implications: for applications requiring tight cylindricity tolerances (e.g., interference-fit shaft holes), operating at the lowest feasible cutting speed (10 m/min in the present range) is preferable. The nonlinear jump in vibration amplitudes between v_c_ = 25 and 30 m/min (2.4–2.6×) strongly suggests proximity to a natural frequency of the drill–spindle–workpiece system. The frequency-domain analysis of the acceleration signals would be valuable for identifying the resonance mode responsible.

Conversely, for applications where Ra is the primary criterion, the intermediate speed of 25 m/min offers the best surface finish. The non-monotonic roughness–feed relationship (Ra peaking at fn = 0.15 mm/rev) warrants further investigation. In conventional turning, surface roughness scales predictably with feed; in drilling, lateral drill vibration, chisel-edge engagement, and chip clogging can disrupt this relationship, particularly at low cutting speeds where built-up edge formation is more probable.

A limitation of the present study is the OFAT design, which prevents the estimation of the interaction effects between the cutting speed and feed rate. A full 3 × 3 factorial experiment with at least two replicates per combination would enable the ANOVA-based decomposition of variance. Additionally, quantified tool wear measurements (flank wear VB per ISO 8688) should be incorporated in future work, as the current conclusion that “no wear was visible” is qualitative only.

Furthermore, the thermal aspects of dry drilling must be considered. Although this study focused primarily on mechanical interactions, significant heat accumulation occurs during dry cutting. According to the recent findings by Zhu et al. [22], temperatures along the cutting edge peak near the drill centre due to the difficult chip evacuation at the core. For conventional HSS tools with a lower thermal stability, this localised heat concentration can cause micro-thermal softening, reducing the drilling stability and potentially increasing the axis drift or geometric errors as the hole deepens. The combined effects of alternative cooling methods (such as MQL) and thermal gradients will be investigated in future stages of this work.

The evolution of the macro-geometrical errors (roundness and cylindricity) is also closely related to the microstructure of the EN-GJS-500-7 ductile iron. As specified in Section 2.3, the material consists of a ferritic–pearlitic matrix with 10% spheroidal graphite nodules. Unlike grey cast iron, where interconnected graphite flakes easily smear to form a continuous solid lubricant film, the spheroidal graphite nodules in ductile iron are isolated within the matrix, preventing the formation of an effective self-lubricating layer under severe dry drilling conditions. During the drilling process, the cutting edges continuously pass through these alternating phases of significantly different hardness (harder pearlite, softer ferrite, and very soft graphite). This periodic transition creates mechanical anisotropy [18] at the micro-scale, inducing micro-fluctuations in the cutting forces [19]. These force variations excite the tool-workpiece system, causing minor dynamic deflections of the HSS drill bit. As a result, the high friction and chip crowding at the core completely dominate the process physics, overshadowing any potential micro-lubricating effect from the graphite nodules, directly leading to increased roundness deviations and cylindricity profile errors as the hole depth increases.

## 5. Conclusions

This study investigated the influence of cutting speed (10, 25, and 30 m/min) and feed rate (0.10, 0.15, and 0.20 mm/rev) on the dimensional accuracy and surface quality of holes drilled in EN-GJS-500-7 ductile cast iron using a standard HSS DIN 338 helical drill. The following conclusions are drawn:

- All drilled holes exhibited dimensional oversize exceeding the IT12 tolerance boundary (PN-EN 22768-1:1999, grade c, ±0.50 mm) for at least some axial positions. The smallest oversize (+0.26 mm for the fitted cylinder) was recorded at v_c_ = 10 m/min, while the largest (+0.57 mm at 10 mm depth) was obtained at v_c_ = 30 m/min. HSS helical drilling in DCI requires subsequent reaming or boring for precision applications.
- Cutting speed is the dominant factor affecting both diameter oversize and cylindricity. Increasing v_c_ from 10 to 30 m/min increased the cylindricity error by 60% (from 0.10 to 0.16 mm) and cylinder diameter oversize by 77% (+0.26 to +0.46 mm). These trends are statistically significant relative to the estimated measurement uncertainty (UR = ±0.021 mm for cylindricity, and ±0.07 mm for diameter).
- Within the tested feed range (0.10–0.20 mm/rev) at a constant v_c_ = 10 m/min, no statistically significant effect on cylindricity or diameter oversize was found; the observed differences fall within the expanded measurement uncertainty. The surface roughness R_a_ showed a non-monotonic dependence on the feed rate, with the highest value (9.1 µm) at f_n_ = 0.15 mm/rev.
- Vibration accelerations increased nonlinearly between v_c_ = 25 and 30 m/min (by a factor of 2.4–2.6×), indicating an approach to a resonance condition of the drill–spindle system. This resonance behaviour is the primary mechanism behind the disproportionate degradation in cylindricity at v_c_ = 30 m/min.
- The best surface finish (R_a_ = 6.6 µm) was achieved at v_c_ = 25 m/min with f_n_ = 0.20 mm/rev. For applications prioritising both the minimum diameter oversize and minimum roughness, v_c_ = 25 m/min and f_n_ = 0.20 mm/rev are recommended within the tested range.
- Future work should employ a full 3 × 3 factorial design with replicated measurements to enable the ANOVA-based separation of the main and interaction effects, and should include a quantified tool wear assessment and frequency-domain analysis of vibration signals.

## Figures and Tables

**Figure 6:**
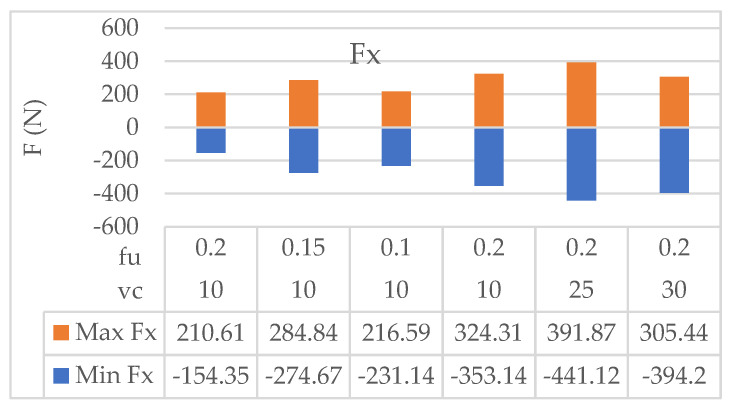
Maximum and minimum values of force *Fx*.

**Figure 7:**
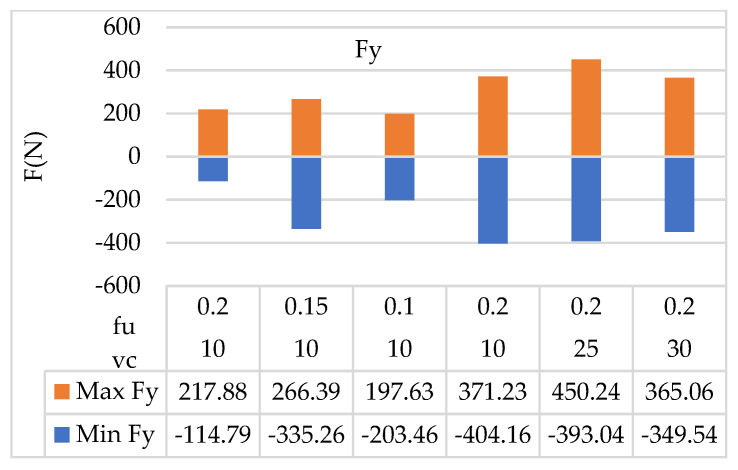
Maximum and minimum values of force *Fy*.

**Figure 8:**
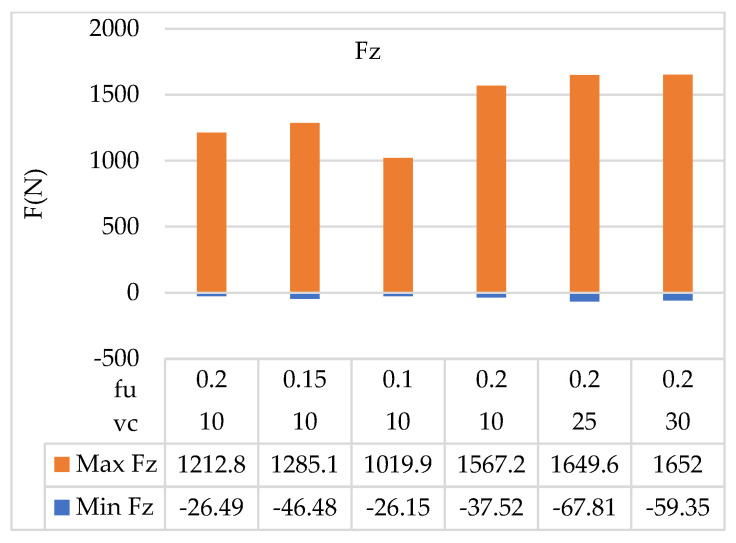
Maximum and minimum values of force *Fz*.

**Figure 9:**
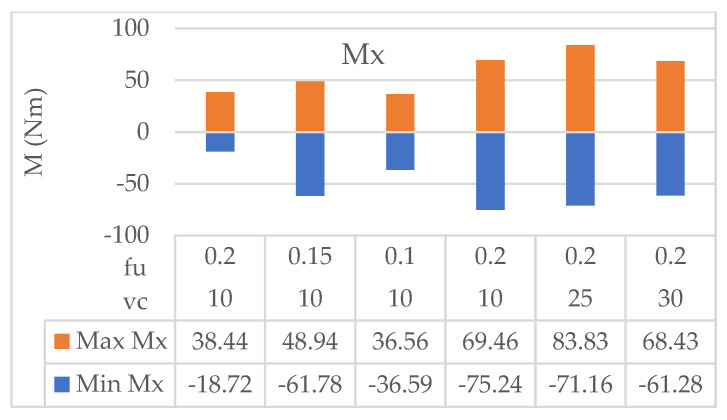
Maximum and minimum values of moment *Mx*.

**Figure 10:**
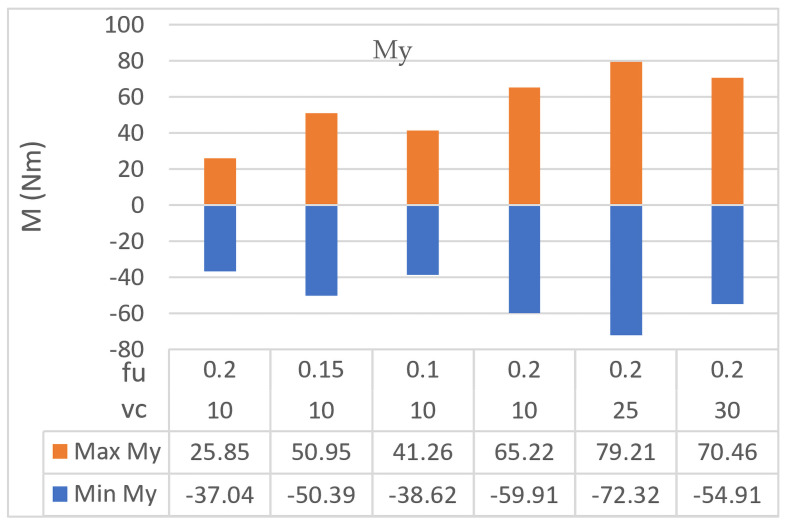
Maximum and minimum values of moment *My*.

**Figure 11:**
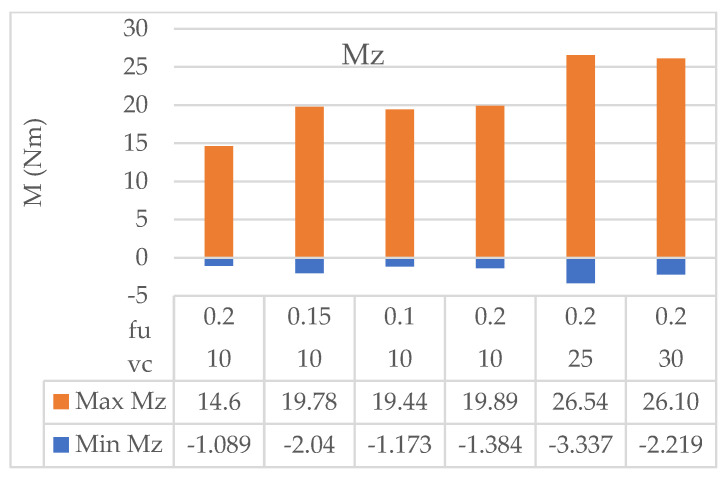
Maximum and minimum values of moment *Mz*.

**Figure 12:**
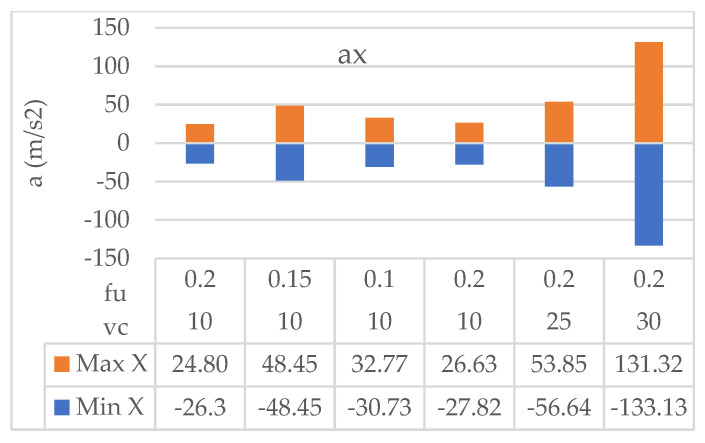
Maximum and minimum values of acceleration ax.

**Figure 13:**
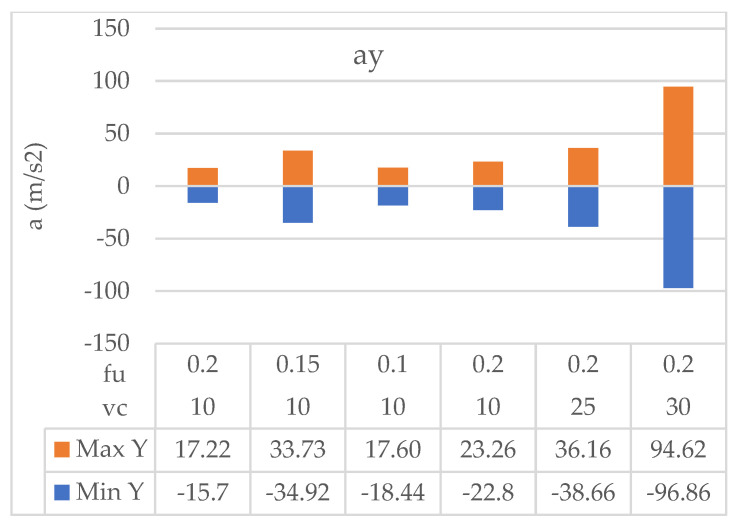
Maximum and minimum values of acceleration ay.

**Figure 14:**
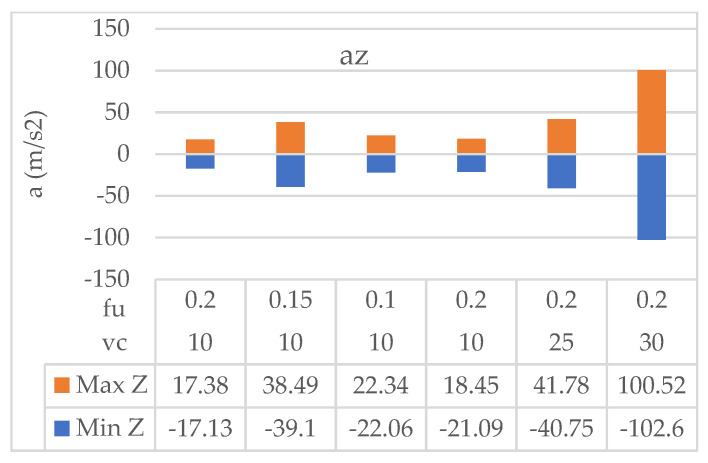
Maximum and minimum values of acceleration az.

**Table 1:** Specimen drilling parameters—OFAT experimental design. v_c_: cutting speed; f_n_: feed per revolution; n: spindle speed; v_f_: feed rate.

Specimen	v_c_ (m/Min)	f_n_ (mm/rev)	n (rpm)	v_f_ (mm/Min)	Series
1	10	0.20	270	54.0	Feed/Speed ref.
2	10	0.15	270	40.5	Feed
3	10	0.10	270	27.0	Feed
4	10	0.20	270	54.0	Repeatability
5	25	0.20	674	134.8	Speed
6	30	0.20	809	161.9	Speed

**Table 2:** Dimensional and technical characteristics of the Ceratizit HSS helical drill.

Technical Data	Value
Drill diameter	11.8 mm
Tool material	HSS
Overall length	142 mm
Working depth	94 mm
Usable length	59 mm
Number of flutes	2
Point angle	118°
Toolmaking standard	DIN 338
Catalogue designation	N.11.80.R.5D.DIN338 VAP

**Table 3:** Chemical composition of EN-GJS-500-7 ductile cast iron (mass %).

C	Si	Cu	Mg	Mn	P	S	Cr	Zn	Fe
3.78	2.46	0.01	0.05	0.32	0.038	0.065	0.031	0.004	Bal.

**Table 4:** Repeatability-based measurement uncertainty (specimens 1 and 4, identical conditions: v_c_ = 10 m/min, f_n_ = 0.20 mm/rev). UR = k·uc, k = 2, coverage probability ≈ 95%.

Parameter	Sp. 1	Sp. 4	|Δ|	Rel. (%)	UR (k = 2)
Ra (µm)	7.5	8.8	1.3	16.5	±0.92 µm
Rq (µm)	10.1	11.0	0.9	8.6	±0.64 µm
Rz (µm)	40.5	43.5	3.0	7.2	±2.12 µm
Cylinder diam. (mm)	12.06	12.16	0.10	0.83	±0.07 mm
Cylindricity (mm)	0.10	0.07	0.03	33.3	±0.021 mm
Mean straightness (mm)	0.045	0.055	0.010	22.2	±0.007 mm

**Table 5:** Maximum recorded values of force, torque, and vibration acceleration for all specimens (read from Figure 6, Figure 7, Figure 8, Figure 9, Figure 10, Figure 11, Figure 12, Figure 13 and Figure 14).

Sp.	Fx_max (N)	Fz_max (N)	Mx_max (Nm)	Mz_max (Nm)	ax_max (m/s^2^)	ay_max (m/s^2^)	az_max (m/s^2^)	Series
1	210.6	1212.8	38.4	14.6	24.8	17.2	17.4	ref.
2	284.8	1285.1	48.9	19.8	48.5	33.7	38.5	Feed
3	216.6	1019.9	36.6	19.4	32.8	17.6	22.3	Feed
4	324.3	1567.2	69.5	19.9	26.6	23.3	18.5	Repeat
5	391.9	1649.6	83.8	26.5	53.9	36.2	41.8	Speed
6	305.4	1652.0	68.4	26.1	131.3	94.6	100.5	Speed

**Table 6:** Surface roughness parameters for all specimens. ISO 4287; evaluation length 5 mm; Gaussian filter λc = 0.8 mm.

Sp.	Ra (µm)	Rp (µm)	Rq (µm)	Rsk (–)	RSm (mm)	Rt (µm)	Rz (µm)
1	7.5	19.5	10.1	0.70	0.250	64.6	40.5
2	9.1	19.9	11.1	0.20	0.302	52.0	41.4
3	7.2	21.9	9.2	0.02	0.268	56.9	41.2
4	8.8	23.5	11.0	0.07	0.333	62.5	43.5
5	6.6	18.3	8.4	0.10	0.326	54.7	34.4
6	7.2	20.5	9.1	0.50	0.307	48.0	38.2

**Table 7:** Measured hole diameters [mm]. Nominal: Ø11.80 mm. Tolerance grade c per PN-EN 22768-1:1999: ±0.50 mm (range 6–30 mm).

Measurement	Sp. 1	Sp. 2	Sp. 3	Sp. 4	Sp. 5	Sp. 6
Diameter at 5 mm	12.13	12.20	12.13	12.18	12.14	12.36
Diameter at 10 mm	12.06	12.27	12.20	12.18	12.20	12.37
Diameter at 15 mm	12.05	12.24	12.16	12.16	12.21	12.29
Diameter at 20 mm	12.04	12.16	12.11	12.17	12.20	12.18
Diameter at 25 mm	12.01	12.11	12.07	12.11	12.08	12.10
Cylinder diameter	12.06	12.20	12.13	12.16	12.17	12.26
Oversize vs. Ø11.80 mm	+0.26	+0.40	+0.33	+0.36	+0.37	+0.46

**Table 8:** Circularity at five axial depths and cylindricity [mm].

Parameter (mm)	Sp. 1	Sp. 2	Sp. 3	Sp. 4	Sp. 5	Sp. 6
Circularity at 5 mm	0.03	0.03	0.02	0.02	0.02	0.02
Circularity at 10 mm	0.02	0.02	0.04	0.03	0.04	0.02
Circularity at 15 mm	0.02	0.02	0.03	0.02	0.03	0.02
Circularity at 20 mm	0.02	0.02	0.02	0.02	0.05	0.03
Circularity at 25 mm	0.03	0.05	0.03	0.02	0.03	0.01
Cylindricity	0.10	0.11	0.10	0.07	0.12	0.16

**Table 9:** Straightness of four axial generators and mean straightness [mm].

Parameter (mm)	Sp. 1	Sp. 2	Sp. 3	Sp. 4	Sp. 5	Sp. 6
Straightness—gen. 1	0.04	0.05	0.04	0.05	0.05	0.05
Straightness—gen. 2	0.04	0.06	0.05	0.06	0.07	0.05
Straightness—gen. 3	0.05	0.06	0.05	0.04	0.05	0.06
Straightness—gen. 4	0.05	0.04	0.07	0.07	0.07	0.06
Mean straightness	0.045	0.053	0.053	0.055	0.060	0.055

## Data Availability

The original contributions presented in this study are included in the article. Further inquiries can be directed to the corresponding author.
